# TORC1-Dependent Phosphorylation Targets in Fission Yeast

**DOI:** 10.3390/biom7030050

**Published:** 2017-07-03

**Authors:** Yoko Otsubo, Akio Nakashima, Masayuki Yamamoto, Akira Yamashita

**Affiliations:** 1 Laboratory of Cell Responses, National Institute for Basic Biology, Nishigonaka 38, Myodaiji, Okazaki, Aichi 444-8585, Japan; otsubo@nibb.ac.jp (Y.O.); yamamoto@nibb.ac.jp (M.Y.); 2 Biosignal Research Center, Kobe University, 1-1 Rokkodai-cho, Nada-ku, Kobe 657-8501, Japan; anakashima@person.kobe-u.ac.jp; 3 Department of Basic Biology, School of Life Science, SOKENDAI (The Graduate University for Advanced Studies), Nishigonaka 38, Myodaiji, Okazaki, Aichi 444-8585, Japan

**Keywords:** TOR complex 1, fission yeast, sexual differentiation

## Abstract

Target of rapamycin (TOR) kinase controls cell metabolism and growth in response to environmental cues such as nutrients, growth factors, and stress. TOR kinase is widely conserved across eukaryotes. As in other organisms, the fission yeast *Schizosaccharomyces pombe* has two types of TOR complex, namely TOR complex 1 (TORC1) and TORC2. It is interesting that the two TOR complexes in *S. pombe* have opposite roles in sexual differentiation, which is induced by nutrient starvation. TORC1, which contains Tor2 as a catalytic subunit, promotes vegetative growth and represses sexual differentiation in nutrient-rich conditions, while TORC2 is required for the initiation of sexual differentiation. Multiple targets of TORC1 have been identified. Some of these, such as S6 kinase and an autophagy regulator Atg13, are known targets in other organisms. In addition, there is a novel group of TORC1 targets involved in the regulation of sexual differentiation. Here, we review recent findings on phosphorylation targets of TORC1 in *S. pombe.* Furthermore, we briefly report a novel *S. pombe* target of TORC1.

## 1. Introduction

It is a fundamental property of cells to grow and decide when to divide by sensing environmental conditions. Target of rapamycin (TOR), a highly conserved serine/threonine kinase, acts as a key factor in this process, and orchestrates various growth-related functions in response to environmental conditions [1]. TOR exists in two distinct complexes termed TOR complex 1 (TORC1) and 2 (TORC2). TORC1 facilitates anabolic processes such as protein synthesis, transcription, and ribosome biogenesis, and inhibits catabolic processes such as autophagy. The macrolide rapamycin, which is known to be an immunosuppressive and antiproliferative agent, acutely and preferentially inhibits TORC1 activity [2]. Mammalian cells have a single TOR kinase, mTOR (mammalian or mechanistic TOR), and both TORC1 and TORC2 share mTOR as the catalytic subunit [3]. In yeast species, TORC1 and TORC2 contain different TOR kinases. In the budding yeast *Saccharomyces cerevisiae*, in which TOR kinase was originally identified [4], TORC1 contains Tor1 or Tor2, and TORC2 contains Tor2 [5]. 

While TORC1-mediated signaling is unambiguously important and indispensable for eukaryotic cells, the current knowledge regarding TORC1 targets is still restricted. The most well-known substrates of TORC1 are S6K1 (p70 ribosomal protein S6 kinase 1), which is a protein kinase to phosphorylate ribosomal protein S6, and 4E-BP1 (eIF4E-binding protein). TORC1 promotes protein synthesis through phosphorylation of these effectors. S6K1 is a member of the AGC (protein kinase A/G/C) kinase family, which includes PKA (protein kinase A, also known as cAMP (cyclic adenosine monophosphate)-dependent protein kinase), AKT (also known as protein kinase B or PKB), SGK (serum- and glucocorticoid-induced protein kinase), and PKC (protein kinase C) [6]. AGC kinases are highly conserved and play an important role in growth, proliferation, and survival. A number of AGC kinases are shown to be activated by phosphorylation. S6K1 is phosphorylated by TORC1 at a threonine residue (Thr389 in human) at the C-terminal hydrophobic motif [7,8]. Phosphorylation of 4E-BP1 by TORC1 leads to its dissociation from eIF4E and alleviates the inhibitory effect of 4E-BP1 on initiation of translation [9,10].

Fission yeast *Schizosaccharomyces pombe* is a unicellular microorganism, which is one of the best model organisms for the study of molecular mechanisms underlying various eukaryotic cellular processes, as well as the budding yeast *S. cerevisiae*. An intriguing feature of *S. pombe* is that sensing of nutritional (especially nitrogen) availability is intimately linked to the change of growth mode from vegetative to reproductive. Under nutrient-rich conditions, the fission yeast normally proliferates by mitotic division in the haploid state, displaying one of two mating types, *h^+^* or *h^−^*. When starved of nitrogen, the cells begin sexual differentiation, that is, cells exit from the G1 phase of the mitotic cycle and conjugate with cells of the opposite mating type to form diploid zygotes, which then undergo meiosis and spore formation. 

**Figure 1:**
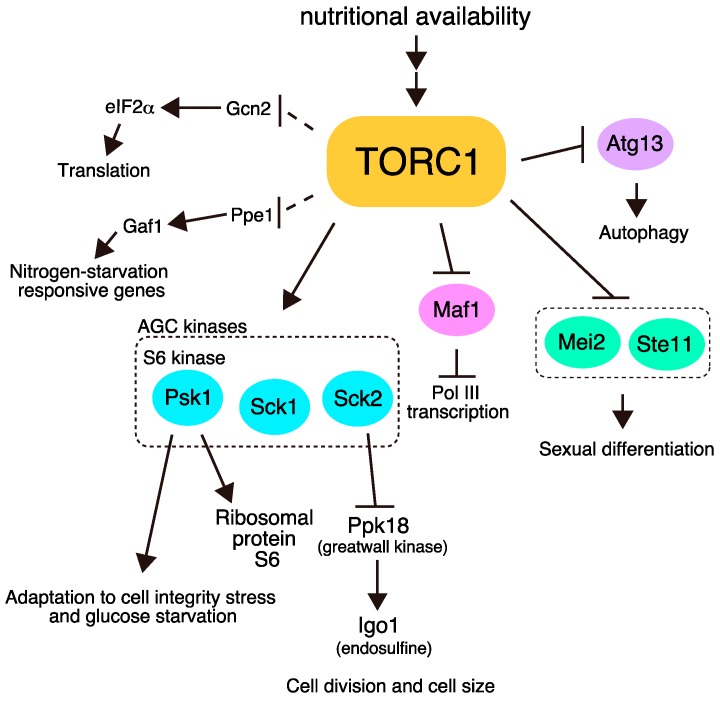
The major downstream pathways of target of rapamycin complex 1 (TORC1) in *Schizosaccharomyces pombe*. TORC1 phosphorylates multiple targets and regulates cell growth and sexual differentiation. Dashed lines represent pathways in which direct phosphorylation by TORC1 has not been demonstrated.

*S. pombe* has two TOR homologs, Tor1 and Tor2 [11,12]. Similar to other eukaryotes, *S. pombe* contains two forms of TOR complex, namely TORC1 and TORC2 [13,14,15]. TORC1, which contains Tor2 as the catalytic subunit, and TORC2, which contains Tor1, play opposite roles in the initiation of sexual differentiation. TORC1 is essential for vegetative growth and represses sexual differentiation under nitrogen-rich conditions [13,14,15,16,17]. Rapamycin does not affect vegetative growth of wild-type *S. pombe* cells [18], although TORC1 is downregulated by treatment of rapamycin [19,20,21,22]. Cells with reduced TORC1 activity, due to a temperature-sensitive *tor2* mutation for instance, stop proliferation and initiate sexual differentiation ectopically even in the presence of ample nitrogen. Conversely, TORC2 is required for sexual differentiation. Cells lacking TORC2 cannot enter sexual differentiation [11,12,23]. It has been demonstrated that the major downstream target of TORC2 is Gad8, an ortholog of AKT [23]. In contrast, although multiple phosphorylation targets of TORC1 have been identified, its critical target(s) remains ambiguous. In this review, we overview TORC1-phosphorylation targets in *S. pombe* (Figure 1).

## 2. Conserved Phosphorylation Targets of TORC1

### 2.1. Psk1

**Figure 2:**
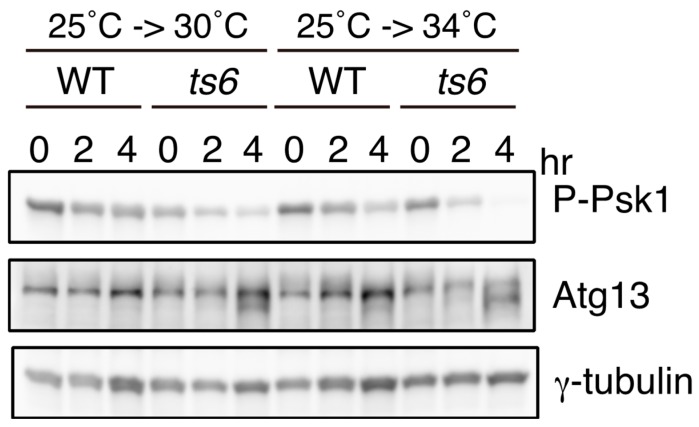
Phosphorylation of Atg13 and Psk1 is regulated by TORC1 in vivo. Phosphorylation of Atg13 and Psk1 in a temperature-sensitive *tor2* mutant. Wild-type (WT; JY3: *h^90^*) and *tor2-ts6* (JT360: *h^90^ tor2-ts6*) cells were grown in YE (Yeast Extract) medium at 25 °C (4 × 10^6^ cells/mL), and then shifted to 30 °C or 34 °C after dilution with the same amount of fresh YE. Cells were disrupted with glass beads in 20% trichloroacetic acid. Cell extracts were subjected to Western blot analysis by using anti-Atg13 antibody (×1000) [24] and anti-phospho-S6 kinase (Thr389) antibody (×1000) (1A5, Cell Signaling Technology, Danvers, MA, USA). Anti-γ-tubulin antibody (GTU-88, Sigma-Aldrich, St. Louis, MO, USA) (×2000) was used as a loading control.

AGC family kinase Psk1 is an S6K1 homolog in *S. pombe* [20]. Similar to mammalian S6K1, several conserved regulatory motifs in Psk1 are phosphorylated by TORC1 and Ksg1 kinase, a homolog of PDK1. Of these target sites, phosphorylation of the hydrophobic motif (Thr415) and the turn motif (Thr392) is regulated by TORC1 [20]. Direct phosphorylation of these two sites by TORC1 has been demonstrated in vitro. Consistent with direct regulation of Psk1 by TORC1, Psk1 is phosphorylated in a nutrient (nitrogen, glucose, and glutamine)-dependent and rapamycin-sensitive manner. Since a commercially available phospho-specific antibody against the phosphorylated hydrophobic motif in mammalian S6K, namely anti-phospho-p70 S6K (Thr389) antibody, can recognize the phosphorylation of Thr415 in Psk1, its phosphorylation status can serve as an excellent readout of TORC1 activity (Figure 2). 

Psk1 phosphorylates ribosomal protein S6, i.e., Rps601 and Rps602, in response to nutritional availability, depending on the TORC1 activity [21]. However, lack of Psk1 does not phenocopy loss of TORC1 function. Whereas the reduction of the TORC1 activity leads to ectopic entry into sexual differentiation, the disruption of the *psk1* gene shows little effect on the growth mode [20]. It has been recently suggested that Psk1 cooperates with TORC2-target Gad8 to adapt to cell integrity stress and glucose starvation, although the precise mechanism remains unclear [25].

### 2.2. Sck1 and Sck2

In addition to Psk1, two other AGC kinases, Sck1 and Sck2, are phosphorylated by TORC1 in vitro [20]. In vivo phosphorylation of Sck1 and Sck2 is indeed dependent on TORC1 [20]. These observations suggest that both Sck1 and Sck2 are direct effectors of TORC1. It was originally shown that both Sck1 and Sck2 have overlapping function with Pka1, which is the catalytic subunit of cAMP-dependent protein kinase (PKA) [26,27]. The interconnection between TORC1 and PKA, both of which regulate the onset of sexual differentiation, by Sck1 has been suggested [28], while the precise relationship between them remains elusive. Recently, it has been demonstrated that TORC1 promotes the activity of the protein phosphatase PP2A-B55 through inhibiting the Greatwall-Endosulfine (Ppk18-Igo1 in *S. pombe*) pathway. PP2A-B55 antagonizes CDK activity in the G2 phase, resulting in increasing cell size in G2 [29,30]. The involvement of Sck2 in the TORC1 and Greatwall-Endosulfine pathway has been suggested, owing to the fact that the overexpression of Sck2 inhibits Greatwall kinase Ppk18 activity [29]. It has also been reported that the overexpression of Sck2 leads to cell elongation [31,32].

### 2.3. Maf1

RNA polymerase III (pol III) generates small untranslated structural RNAs for protein synthesis, such as 5S rRNA and tRNA. In response to nutrient availability and cellular stress, pol III transcription is controlled by a conserved repressor Maf1, which is regulated by phosphorylation [33,34,35]. Dephosphorylated Maf1 represses pol III-dependent transcription. In the budding yeast *S. cerevisiae*, the major Maf1 kinase is Sch9, which is an ortholog of S6K1/Psk1 and a target of TORC1 [36,37], whereas *S. cerevisiae* TORC1 could weakly phosphorylate Maf1 in vitro [38]. In mammalian cells, TORC1 is responsible for Maf1 phosphorylation [39,40,41]. In *S. pombe*, Maf1 phosphorylation depends on TORC1, but not TORC2, although it remains elusive whether TORC1 is directly involved [22].

### 2.4. Atg13

Autophagy is a highly conserved intracellular degradation mechanism in eukaryotes [42]. TORC1 negatively regulates autophagy under nutrient-rich conditions. In the budding yeast *S. cerevisiae*, the conserved serine/threonine kinase Atg1 is pivotal in the regulation of autophagy, which forms a multimeric protein complex [43,44,45]. Atg13 is an essential regulatory member of the Atg1 kinase complex. In *S. cerevisiae*, Atg13 is highly phosphorylated in nutrient-rich conditions, and becomes rapidly dephosphorylated upon nutrient deprivation or inhibition of TORC1 by rapamycin [43]. It has been shown that TORC1 directly phosphorylates Atg13 at multiple serine residues in vitro [46]. TORC1 inhibition, which leads to dephosphorylation of Atg13, promotes Atg1 complex formation, increases Atg1 activity, and results in the induction of autophagy [43,46]. Since Atg1 also contains a phosphorylation site that is downregulated upon rapamycin addition [47], Atg1 might also be a substrate of TORC1 in *S. cerevisiae*.

In mammals, TORC1 also negatively regulates autophagy [48]. Mammalian homologs of Atg1 are uncoordinated-51-like kinase 1 and 2 (ULK1 and ULK2). As in *S. cerevisiae*, ULK1 forms a multimeric protein complex, including mammalian Atg13 [49,50,51]. While TORC1 has been shown to phosphorylate both ULK1 and Atg13 in vitro [50,51], TORC1 has no impact on the formation of the ULK1 complex [50,52]. In vivo phosphorylation of ULK1 by TORC1 prevents the interaction between ULK1 and AMP-activated protein kinase (AMPK) [53]. It has also been shown that TORC1 phosphorylates an autophagy regulator Atg14 in mammalian cells [54].

*S. pombe* carries an autophagy system similar to that of other eukaryotes. Autophagy-deficient mutants lose their viability under conditions of prolonged nitrogen starvation [24]. In addition, these mutants are unable to initiate sexual differentiation under nitrogen starvation conditions [24]. As in *S. cerevisiae*, it has been demonstrated that *S. pombe* Atg13 is highly phosphorylated under nutrient-rich conditions, and this phosphorylation decreases upon nitrogen starvation, which induces the inactivation of TORC1 [24]. Here, we present data to confirm that the phosphorylation of Atg13 is dependent on TORC1, as shown using a temperature-sensitive *tor2* mutant (Figure 2). In *tor2-ts6* cells, Atg13 migrated faster when cells were shifted to a restrictive temperature of 30 °C or 34 °C, indicating that the phosphorylation of Atg13 is dependent on TORC1. This also implies that Atg13 serves as a good readout of TORC1 activity, as is the case with Psk1. While the phosphorylation of *S. pombe* Atg1 is also alleviated upon nitrogen starvation [24], it remains unknown whether Atg1 is a TORC1 target.

## 3. Direct Phosphorylation Targets of TORC1 that Regulate Sexual Differentiation

An RNA-binding protein, Mei2, is a master regulator of meiosis in *S. pombe* [55,56,57]. In addition to the regulation of meiosis, Mei2 functions in the earlier stages of sexual differentiation, and the loss of Mei2 results in a partial deficiency in G1 arrest and conjugation [55,58]. Mip1, which is the *S. pombe* ortholog of mammalian Raptor, an essential component of mTORC1, was originally isolated as a genetic interactor of Mei2 and was shown to physically interact with Mei2 [59]. Physical interaction between Mei2 and Tor2 has also been demonstrated [13]. These observations suggested the close relationship between Mei2 and TORC1, and it has indeed been demonstrated that TORC1 phosphorylates multiple sites of Mei2 in vivo and in vitro [58]. Mei2 phosphorylation by TORC1 induces polyubiquitination and proteasomal degradation of Mei2, leading to the suppression of sexual differentiation [58]. Mei2-like protein is widely conserved in plants [60,61]. It is intriguing that an *Arabidopsis* Raptor homolog, named AtRaptor1B, binds to Mei2-like protein AML1 as in *S. pombe* [60], suggesting a conserved regulatory mechanism between *S. pombe* and plants.

**Figure 3:**
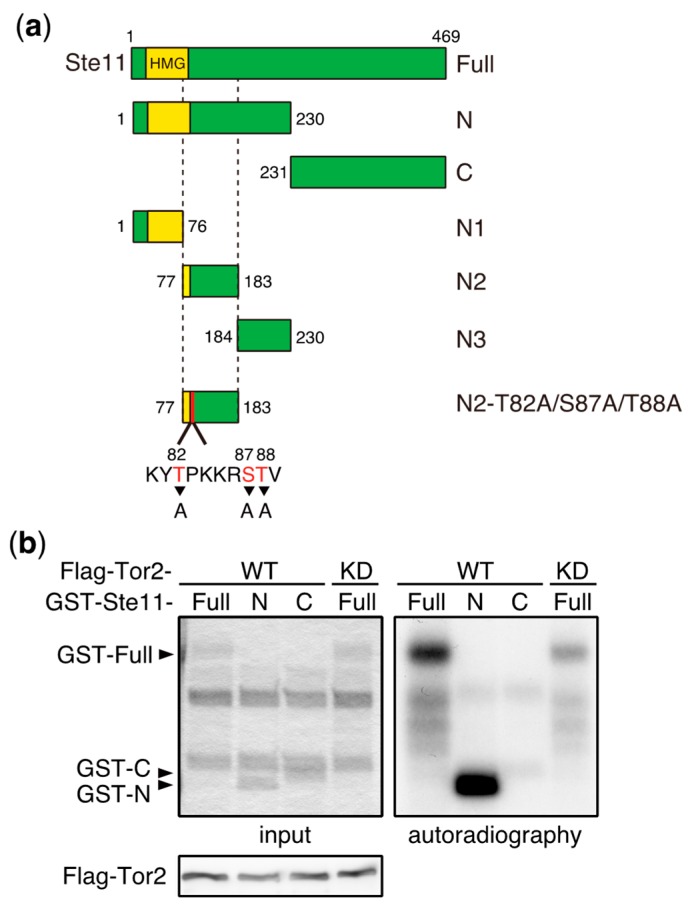

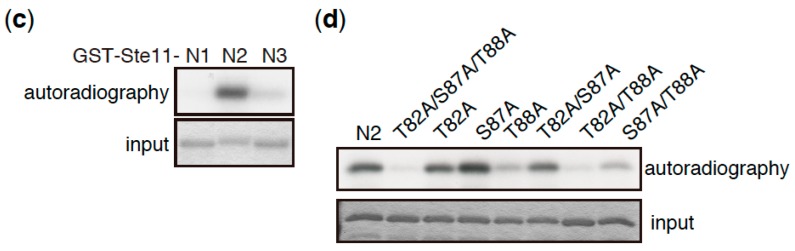
Ste11 is phosphorylated by TORC1. (**a**) Schematic illustration of truncation series of Ste11 used in in vitro kinase assays. HMG represents a DNA binding domain, HMG (high mobility group) box. (**b**–**d**) In vitro phosphorylation of Ste11 by TORC1. Flag-tagged Tor2 was immunoprecipitated from *S. pombe* cell extracts using an anti-FLAG antibody. The same immunoprecipitation conditions as described in our previous study were used, in which other components of TORC1 were co-precipitated [15]. The kinase preparation was then incubated with a GST (Glutathione S-transferase)-Ste11 fusion protein, which harbors Ste11 residues 1–469 (Full), 1–230 (N), 231–469 (C), 1–76 (N1), 77–183 (N2), and 184–230 (N3), in the presence of radioactive ATP. Tor2 phosphorylated GST-Ste11-Full and Gst-Ste11-N efficiently, whereas a kinase-dead form of Tor2 (KD) displayed only marginal phosphorylation toward GST-Ste11-Full (**b**). The exogenously expressed kinase-dead Tor2 in wild-type cells may form a heterodimer with endogenous wild-type Tor2, acquiring a weak activity, as in other organisms [66,67]. Ste11-N2 was also phosphorylated efficiently (**c**). An alanine substitution series in Ste11-N2 indicated that the major phosphorylation residue was Thr88, while Thr82 was also phosphorylated, but less efficiently (**d**).

Whereas TORC1 phosphorylates and induces the degradation of Mei2, the deletion of the *mei2* gene only partially suppresses the phenotype of *tor2* mutant cells, which initiate sexual differentiation under nutrient-rich conditions [58]. This suggests that Mei2 is not the sole TORC1 target that regulates the initiation of sexual differentiation. Consistent with this idea, we found that Ste11, a major transcription factor controlling the switch from cellular proliferation to sexual differentiation in *S. pombe*, is phosphorylated by TORC1 (Figure 3). Ste11 activates many genes required for the initiation of sexual differentiation, including *mei2* [62,63,64]. In vitro kinase assays have demonstrated that the N-terminal fragment of Ste11 (N2, residues 77–183) was phosphorylated by immunoprecipitated TORC1 (Figure 3a–c). An analysis of alanine substitution mutants indicated that Thr88 was a major phosphorylation residue on Ste11, and Thr82 was also phosphorylated, although less efficiently (Figure 3d). It has been shown that the inactivation of TORC1 causes nuclear accumulation of Ste11 [65], suggesting that TORC1 regulates nuclear localization of Ste11 by phosphorylation. Future studies may shed light on the significance of Ste11 phosphorylation by TORC1 with regard to the regulation of sexual differentiation.

## 4. Indirect Effectors of TORC1

### 4.1. Ribosomal Protein S6

Ribosomal protein S6 (Rps6) is an evolutionarily conserved protein in eukaryotes and is involved in many cellular processes [68]. In *S. pombe*, Rps6 is encoded by two genes, *rps601* and *rps602* [21]. Rps6 is phosphorylated at conserved Ser235 and Ser236 residues by Psk1, a S6k1 homolog, when TORC1 is active [20,21]. The anti-phospho-Akt substrate (PAS) antibody can be used to detect the phosphorylation of Rps6 at these sites [21]. It has been shown that the other TORC1 effector Sck2 is also involved in Rps6 phosphorylation at these residues in a medium-dependent manner, although it is unknown whether Sck2 directly phosphorylates Rps6 [32]. AGC kinase Gad8, which is the major effector of TORC2, has also been shown to contribute to Rps6 phosphorylation [22]. Since the phosphorylation of Rps6 at Ser235 and Ser236 is dispensable for cell viability [21], their phosphorylation might play a role(s) in particular conditions.

### 4.2. Ppk18 and Igo1

TORC1 negatively regulates the Greatwall-Endosulfine, Ppk18-Igo1, via the inhibition of the Greatwall kinase Ppk18 as mentioned above. The downregulation of Ppk18 has been suggested to be mediated by Sck2, which is a direct effector of TORC1, since it was observed that Sck2 overexpression inhibits Ppk18 activity [29]. It has also been demonstrated that Ppk18-dependent phosphorylation of Igo1 is induced by the reduction of TORC1 activity in *tor2* mutant cells, or by the treatment with TOR inhibitors such as rapamycin and Torin1 [29].

### 4.3. eIF2α

The phosphorylation of eukaryotic initiation factor 2 alpha (eIF2α) causes repression of general protein synthesis in response to various forms of environmental stress. Gcn2 is known to phosphorylate eIF2α in mammals and in the budding yeast *S. cerevisiae* [69]. In *S. cerevisiae*, TOR prevents the phosphorylation of eIF2α by Gcn2 [70,71]. Similarly, in *S. pombe*, Gcn2 phosphorylates eIF2α at serine 52 under TORC1-inactive conditions such as mutation of Tor2 and nitrogen deprivation, while the precise mechanisms underlying Gcn2 activation remain unknown [72]. Serine 52 phosphorylation of *S. pombe* eIF2α can be detected by the commercially available anti-phospho-eIF2α antibody [72].

### 4.4. Gaf1

*S. pombe* TORC1 promotes the phosphorylation of the GATA transcription factor Gaf1 by inhibiting the PP2A-like phosphatase Ppe1 [73]. Phosphorylated Gaf1 is retained in the cytoplasm and does not exert its activity as a transcription factor. When TORC1 is inactivated, Gaf1 is dephosphorylated through the activation of Ppe1 phosphatase and translocates into the nucleus. Thus, the localization of Gaf1 can be a readout for TORC1 activity. Gaf1 positively regulates early response genes under nitrogen stress, such as the 2-oxoglutarate-Fe(II)-dependent oxygenase *isp7*, and negatively regulates later nitrogen stress genes, such as *ste11* [73,74]. The regulation of GATA transcription factors downstream of TORC1 is conserved in *S. cerevisiae* [75,76,77].

## 5. Conclusions

In this review, we have overviewed the *S. pombe* TORC1-dependent phosphorylation targets (Figure 1). Recent studies in *S. pombe* have revealed a variety of TORC1-related regulatory mechanisms. In particular, *S. pombe* TORC1 (and TORC2) regulates the change of growth mode in response to environmental conditions, which could be regarded as a simple model of cell differentiation. Hopefully, *S. pombe* research in the future will bring a new perspective to the study of TOR-mediated cellular regulation.

Since the disruption of *gad8*, which is phosphorylated and activated by TORC2, results in the same phenotype as the lack of TORC2, such as temperature sensitivity and sterility, Gad8 is thought to be the major TORC2 phosphorylation target [23]. In contrast, the deletion of any TORC1 phosphorylation target fails to phenocopy the TORC1 mutant, suggesting that the key target(s) of TORC1 remains to be identified; nonetheless, we cannot rule out the possibility that TORC1 has multiple important targets and that simultaneous deletion of such factors is required to induce the phenotypes of the TORC1 mutant. In either case, further investigation of TORC1 targets in *S. pombe* will contribute to the clarification of the essence of the TOR signaling pathway.
